# Longitudinal Preceptor Assessment of Entrustable Professional Activities Across Introductory and Advanced Pharmacy Practice Experiences

**DOI:** 10.3390/pharmacy13030072

**Published:** 2025-05-21

**Authors:** Jennie B. Jarrett, Abigail T. Elmes-Patel, Sheila M. Allen, Marlowe Djuric Kachlic, Allison E. Schriever, Tara P. Driscoll, Ara Tekian, Jeffrey J. H. Cheung, Edward Podsiadlik, Stuart T. Haines, Alan Schwartz

**Affiliations:** 1 Department of Pharmacy Practice, Herbert M. and Carol H. Retzky College of Pharmacy, University of Illinois Chicago, Chicago, IL 60612, USA; jarrett8@uic.edu (J.B.J.); sallen7@uic.edu (S.M.A.); mdjuri1@uic.edu (M.D.K.); aes@uic.edu (A.E.S.); tpd4@uic.edu (T.P.D.); 2 Department of Medical Education, College of Medicine at Chicago, University of Illinois, Chicago, IL 60612, USA; tekian@uic.edu (A.T.); jcheung@uic.edu (J.J.H.C.); alansz@uic.edu (A.S.); 3 College of Education, University of Illinois Chicago, Chicago, IL 60612, USA; epodsi1@uic.edu; 4 School of Pharmacy, University of Mississippi, University, MS 38677, USA; sthaines@olemiss.edu

**Keywords:** workplace-based assessment, advanced pharmacy practice experiences, competency-based education, entrustment–supervision scales, entrustable professional activities

## Abstract

The objective was to evaluate the growth in pharmacy student performance in entrustable professional activity (EPA) assessments across the experiential curriculum based on preceptor assessments on an entrustment–supervision (ES) scale. This retrospective cohort study used assessments based on the 2016 American Association of Colleges of Pharmacy Core EPAs and an expanded ES scale during introductory and advanced pharmacy practice experiences (IPPEs/APPEs) in the third and fourth professional years from fall 2020 to fall 2023. The primary outcome was the change in ES level, assessed by preceptors over time. The secondary outcomes were growth rates across types of experiences, training environments, and experience order. A conditional growth curve model and ordinal mixed effects model were used to demonstrate discrete entrustment decisions. A total of 509 students received 12,426 assessments by 557 preceptors. Raw ES levels and unconditional growth curves for EPA show increases in entrustability from years P3 to P4. Comparing care settings, there was lower entrustment in inpatient than outpatient settings and at academic medical centers than other settings. There were no significant differences in ES levels regardless of which IPPE was taken first. However, when the first APPE was an inpatient medicine experience, ES levels across APPEs for EPA 3 were higher when compared to ambulatory care as the first APPE, and they were higher for EPA 5 when compared to community pharmacy as the first APPE. Paired with ES scales, EPAs can be integrated into pharmacy experiential curricula to demonstrate longitudinal growth in student entrustment.

## 1. Introduction

While there remain significant logistical barriers to the full implementation of competency-based education (CBE), principles of competency-driven and outcome-focused education have been developed and implemented within pharmacy education and are recognized within accreditation standards [1,2,3,4,5,6,7].

Entrustable professional activities (EPAs) are an important component of CBE. While EPAs are descriptors of the work a professional performs [8], they go beyond a list of competencies that describe knowledge, attitudes, and skills used within the field by describing the discrete and essential activities a professional is expected to masterfully perform [6]. The role of a health professional includes a set of EPAs they would be expected to perform in their job [8,9]. Educators can use EPAs in two ways: “(1) as a link between individual competencies for mastery and professional responsibilities in practice and (2) as a mechanism for preceptors to assess the student’s progression using levels of decreasing supervision” [8].

EPAs were originally used as an assessment mechanism in health profession education. The performance of a student in an EPA is assessed by the level of supervision required in an authentic workplace, rather than assigning a score, percentage, or letter grade typically used when rating student performance in traditional academic coursework [9,10]. Such an assessment represents the inverse relationship between supervision and entrustment provided by the preceptor when a learner performs a task [11]. As a learner builds their knowledge, attitudes, and skills, their need for supervision decreases [8]. However, entrustment decisions likely vary based on the settings of care, patient complexity, the activity to be performed, and limitations imposed by licensing bodies.

Developed as a component of a curricular transformation in 2016–2017, the University of Illinois Chicago Retzky College of Pharmacy (UIC) Experiential Education curriculum is made up of a nine-semester course series of skills-based lab courses and clinical rotations in accordance with the Accreditation Council for Pharmacy Education (ACPE) Standards 2016 [5]. Introductory Pharmacy Practice Experiences (IPPEs) are utilized to introduce and incorporate students into a workplace-based learning experience within common pharmacy practice models. They are sequenced to prepare students for advanced pharmacy practice experiences (APPEs) and are often preceded by skills-based lab simulations to enhance student knowledge and learning prior to workplace-based learning experiences [5]. The APPE curriculum builds on the foundational knowledge, skills, and attitudes gained from the IPPEs to advance responsibilities across patient care areas, including the four required areas of community pharmacy, ambulatory patient care, hospital/health system pharmacy, and inpatient general medicine patient care [5]. Assessments for experiential courses are individualized by each college of pharmacy but are required to provide formative feedback during the experience. Competence is assessed minimally at the midpoint and end of a rotation [5]. EPAs were integrated throughout the UIC experiential curriculum starting in 2019 as an assessment for the IPPE and APPE courses and as a reflective component for students in the skills-based lab courses. Table A1 denotes the experiential courses, their descriptions, and the utilization of EPAs.

The IPPE and APPE components of the experiential curriculum are workplace-based learning requirements, and thus the EPA framework naturally works for assessment across the course series. The core EPAs for pharmacy graduates are a broad set of work tasks expected of a pharmacist across pharmacy practice areas, and thus EPAs were mapped for each type of IPPE and APPE (Table A2).

Entrustment–supervision (ES) scales provide students with formative feedback and a summative assessment of their performance for each EPA. The utility of traditional ES scales for assessment in pharmacy experiential curricula note overestimation by preceptors as well as students for self-reflection [12,13]. The Jarrett ES scale (Table 1), based on the Chen ES scale used in medical training and translated for use in pharmacy education, was selected for use by the UIC to assess learners during IPPEs and APPEs [14,15].

This scale provides additional sublevels to the traditional ES scale, which permits more precise assessment of competence and performance at lower ES levels [14,15]. To accurately assess student performance and decrease overestimation, raters were only permitted to assign learners rating no higher than Level 2b in first- and second-year student IPPEs, Level 3a in third-year student IPPEs, and Level 4 in fourth-year student APPEs on the Jarrett ES scale. The objective was to evaluate the growth in pharmacy students’ EPA performance using an ES scale across a pharmacy experiential curriculum. The key research question was whether EPA-based assessments could show longitudinal growth from IPPEs to APPEs.

## 2. Materials and Methods

A retrospective cohort design was utilized to evaluate the growth in pharmacy students’ performance on EPA-based assessments across the UIC experiential curriculum using preceptor evaluations related to core EPAs [16].

### 2.1. Study Population and Recruitment

Pharmacy students enrolled in the Doctor of Pharmacy (PharmD) program at UIC were eligible for inclusion. UIC pharmacy students were included if they completed any of the IPPEs or APPEs that use the EPA-based assessments. The targeted study population was the UIC PharmD graduating class of 2022 since this class was the first class to be required to use the EPA assessment framework across all IPPEs and APPEs. Assessment data was included for students in the UIC graduating classes of 2023 and 2024 who completed IPPE assessments. No students who completed IPPE and APPE assessments were excluded.

### 2.2. Data Collection

Preceptors submitted all learner assessments on the Jarrett ES scale electronically through eValue via the MedHub^®^ (Minneapolis, MN, USA) online portal. Table A2 notes EPAs assessed during each IPPE and APPE. The ES levels from final assessments from preceptors for the EPAs were collected from eValue for UIC PharmD students in courses PHAR 515 and 516 and each APPE for the EPAs corresponding to the rotation type.

### 2.3. Outcomes

The primary outcome was developmental growth based on ES ratings of pharmacy students over time, as assessed by preceptors. Secondary outcomes were the differences in ES ratings across different types of experience (community, hospital, ambulatory care, inpatient general medicine); across different training environments (academic medical centers [AMC] versus other types of health care environments [non-AMC]; and the order of the practice experience.

### 2.4. Data Analysis

For the primary outcome, a conditional growth curve model was used, similarly to Schwartz et al. [17]. An ordinal mixed-effects model was used to demonstrate the discrete decisions made for entrustment, and a linear mixed-effects model was fitted for improved visualization [17]. Table A3 denotes the mapped response levels for the ordinal and linear mixed-effects models. Models were fitted separately to each EPA under a generalized additive model framework using R 4.2 (R Core Team, Vienna, Austria) and the mgcv package 1.8-41 [18]. All models included fourth-degree polynomial functions of time as fixed-effects random intercepts, time slopes, and a random intercept for preceptors. One conditional growth model for each EPA also included the fixed effects of relevant rotation characteristics (core vs. elective, clinical vs. non-clinical, AMC vs. non-AMC, and inpatient vs. outpatient) and interactions between those characteristics and the fourth-degree polynomial time components. A second model added a fixed effect of the student’s first IPPE rotation (PHAR 515 vs. PHAR 516) and the interaction between that effect and the polynomial time components, and this was applied only to assessments provided on rotations in the following year. A third model added a fixed effect of the student’s first APPE rotation in their fourth year and the interaction between that effect and the polynomial time components, and this was applied only to assessments on subsequent fourth-year rotations. The statistical difference was determined significant at *p* < 0.05 between variables.

## 3. Results

ES ratings from a total of 509 students were included in the analysis. The demographic information for these students can be found in Table 2.

The included students received 12,426 ES ratings by 557 preceptors during the study period. The number of ES assessments by graduating class and by preceptors for each included EPA can be found in Table A4.

**Figure 1:**
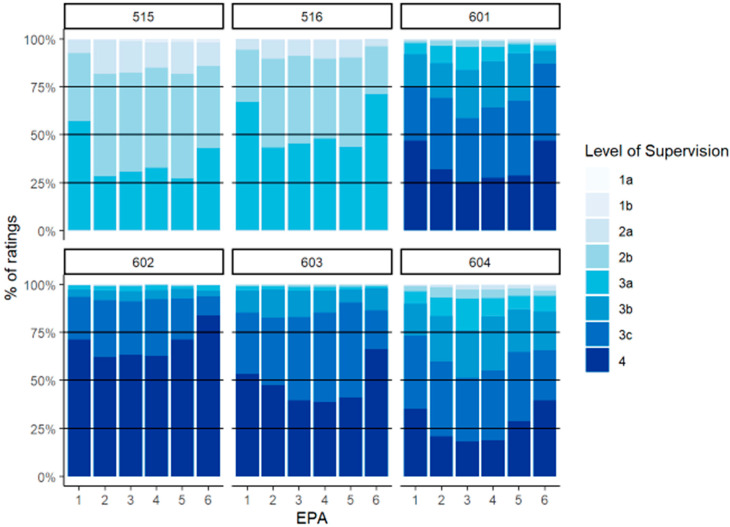
Distribution of preceptor assessments by course in third (PHAR 515/516) and fourth (PHAR 601–604) years.

The distribution of preceptor assessments by experiential course can be seen in Figure 1. The predominant ES levels in the IPPE (PHAR 515 and 516) course were 2b and 3a, with a greater distribution of 3a assessments in PHAR 516 (ambulatory care). For the APPEs, which could be taken in any order, the highest percentage of Level 4 assessments was in PHAR 602 (community) and the lowest percentage of Level 4 assessments was in PHAR 604 (inpatient medicine).

**Figure 2:**
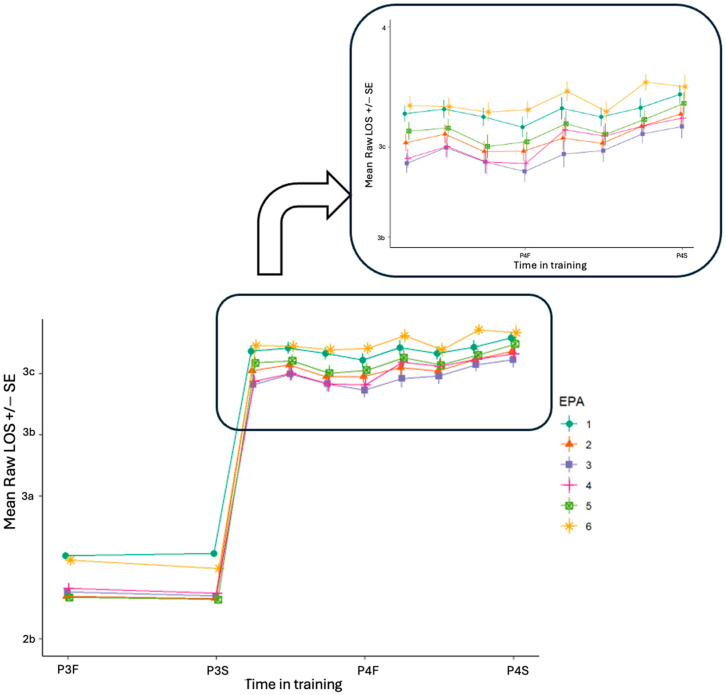
Raw mean level of supervision (LOS) over time by EPA assessed by preceptors. The inset zooms in on the fourth year, with each EPA shown as a different color across the two years.

**Figure A1:**
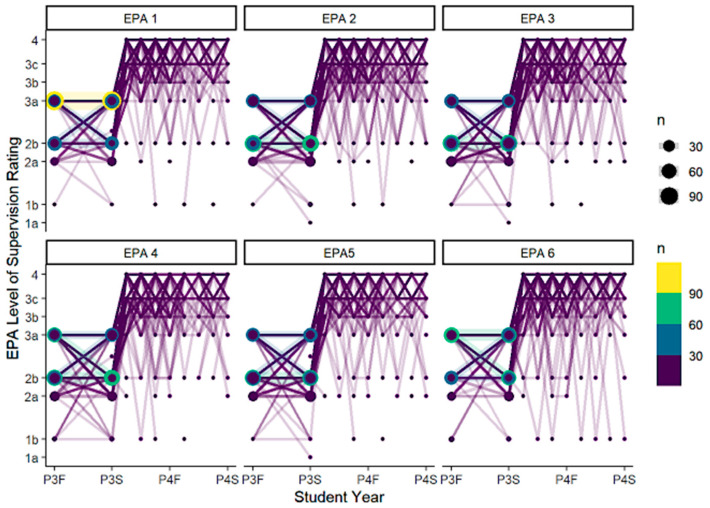
Distribution of preceptor assessments of students over time by EPA.

By EPA, there is a clustering of ES ratings in years P3 and P4, the distribution of which can be found in Figure A1. The raw ES levels over time by EPA show increases in entrustability for all EPAs over time (Figure 2).

Specifically, Figure 2 shows the raw ES levels from preceptors across the experiential curriculum, where each line characterizes the mean growth in each EPA. During the P4 year, the raw ES levels show that EPA 6 had the highest level of entrustment consistently across the year when compared to EPAs 1 through 5. Conversely, EPA 3 had the consistently lowest ES ratings compared to other EPAs across the year.

### 3.1. Unconditional Growth Curve by Preceptor Assessment

**Figure 3:**
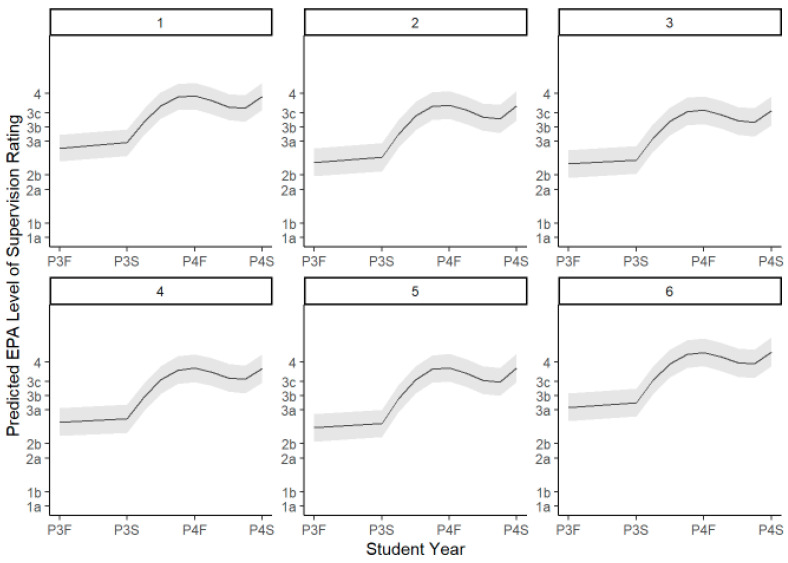
Unconditional growth curves by preceptor assessment and EPA with 95% CI.

The unconditional growth curves by preceptor assessment show growth over time for students from year P3 to P4 across all six EPAs (Figure 3).

All components of the time polynomial were statistically significant; there was a significantly positive linear slope, a negative quadratic term, a negative cubic term, and a positive quadratic term for each EPA. While there was, as seen by visual inspection, a slight dip in ES levels across all EPAs at the midpoint of fourth year, this was not a statistically significant change.

### 3.2. Conditional Growth Modeling for Preceptor Assessment

**Figure 4:**
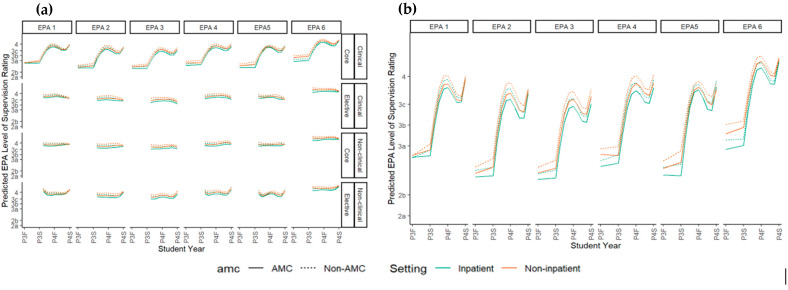
Conditional growth curves. (**a**) Comparison across core and elective experiences and clinical versus non-clinical experiences; (**b**) the difference in growth in core clinical experiences between setting and training environments.

Figure 4 shows the conditional growth models used to determine growth differences across different types of experiences and different training environments.

Measurable growth was only seen in the clinical core rotations (PHAR 601 and 604). When comparing assessments based on care setting for inpatient to outpatient clinical core experiences, there was a difference in ES level, with lower entrustment in the inpatient setting than outpatient setting across all EPAs (ordinal and linear model *p* < 0.05 for each EPA). Similarly, there was a difference in the ES level at AMCs when compared to non-AMCs across all EPAs, with lower entrustment at AMCs (ordinal model *p* < 0.001 for each EPA, linear model *p* < 0.02 for each EPA; Table A5). For EPA 6, a difference in slope was detected when comparing inpatient and outpatient curves (*p*-value < 0.05 in ordinal or linear model). No other differences in slope were noted.

In models based on IPPE or APPE order, there were no significant differences in P4 ES levels regardless of which P3 IPPE was taken first by the student. However, when the first APPE was PHAR 604 (inpatient medicine), the overall ES levels for EPA 3 over the rest of the P4 rotations were higher (less supervision required) than when the first APPE was PHAR 601 (ambulatory care). Similarly, ES ratings were higher for EPA 5 when the first APPE was PHAR 604 (inpatient medicine) when compared to PHAR 602 (community).

## 4. Discussion

EPAs using ES ratings are an authentic way to assess learners over time, particularly in health professions’ education [8]. The results note clear growth in performance across IPPEs and APPEs measured with the Jarrett ES scale as well as the margin of variation in performance. While there is evidence in pharmacy education to support the implementation of EPAs for assessment at points in time during the IPPEs and APPE curricula [12,13,19,20], this study is the first to show growth over time from a broader, continuous curricula covering IPPE to APPE. The direct patient care EPAs (numbers 1 through 5) in our dataset show lower ES levels (more supervision needed) early in the APPE year with linear growth, compared to non-direct patient care EPAs (EPA 6) with higher ES levels documented early in year P4. Our data is the first to link IPPE assessments within this trajectory and show that in direct and non-direct patient care EPAs this starts before the final year in the curricula.

Our data failed to show any growth in ES ratings during non-clinical and elective APPEs over the fourth year. ES levels were consistently higher during these experiences. This may be due to prior experience through work outside of pharmacy school or higher student motivation and interest in a specialty area where they took didactic elective courses. Alternatively, this finding may be a function of the expectations of a student’s preceptors at that point in the curriculum rather than based on direct observations. The EPAs most related to the Pharmacists’ Patient Care Process are noted to be the most important when determining practice-readiness, particularly in clinical areas [21]. Further, each student is expected to meet the recommended Level 3 on an ES scale before graduation [22]. These results show that using summative entrustment decisions is feasible as the high proportion of students at the expected ES level aligns with historical assessment scores across IPPEs and APPEs used to evaluate practice-readiness at the UIC.

The Jarrett ES scale allowed for more discrete recognition of growth. From a preceptor perspective, this lowering of rating, while not statistically different, may be used to identify burnout during a busy clinical year, or it may signal fewer observations on a rotation during a time when students are interviewing for residencies or fellowships, lowering their confidence in trusting a student at a higher level.

### 4.1. Variation in Growth and Achievement

#### 4.1.1. By Training Environment

This work highlights the differences in entrustment over time in different training environments. The distribution of ES levels across the courses highlights that less supervision is perceived to be needed in settings where decisional authority is lower, such as community pharmacies (PHAR 602 and 603), when compared to inpatient or ambulatory care clinics (PHAR 601 and 604), where decisional authority is greater and the analysis of complex medical information is needed. Further, pharmacists must obtain licensure for practice to authorize the distribution of medication. This lack of ability to practice with indirect supervision for an EPA makes it difficult to provide opportunities for authentic autonomy in this type of pharmacy practice. Yet, preceptors consistently provided Level 4 assessments for many students, similarly to previous research [19]. This inflation may indicate a gap in training and lend itself to patient safety concerns that a pharmacist will not be prepared to practice in this area when they are licensed [23].

Differences were seen in the clinical areas where inpatient rotations had lower entrustment than outpatient settings for EPAs 1–6. Inpatient patient care, particularly at AMCs, may represent a higher acuity and complexity level for pharmacy practice, due to the higher prevalence of high-risk medications that may cause patient harm [24]. However, the difference is approximately 0.1 compared to outpatient APPEs, and it is unclear if this difference is meaningful.

#### 4.1.2. By Rotation Order

The results show that earlier clinical inpatient rotations had higher ES levels over time according to preceptor assessments. These types of rotations are often of an advanced caliber with more complex patients and a higher patient volume. Cognitive load theory describes the capability of a learner to perform a function based on their cognitive load with their working memory [25]. Activities in clinical spaces, particularly inpatient environments and at AMCs, may show higher performance over time due to the implications of intrinsic cognitive overload due to increased patient complexity and higher decisional authority [25]. Early exposure to clinical training in health profession education is shown to improve student satisfaction and clinical performance [26,27]. Clinical rotations are an excellent way for students to access this early exposure, but the APPE timepoint in pharmacy curricula may be too late for the benefits to fully come to fruition and may hinder foundational learning, impacting performance over time, as noted by these results. Similarly to differences in entrustment by training environment, these differences are small (<0.5) on the ES scale, and it is unclear if this is meaningful. However, this may be useful in developing individualized student schedules by placing these types of rotations earlier or later depending on a student’s needs.

### 4.2. Limitations

While the cohort included over 500 students and 12,000 preceptor ratings, most data, particularly the APPE assessments, were from a single pharmacy school cohort. While this class was like other classes demographically, student differences may not be fully understood and could have influenced the results. Students were not able to select the rotation order but were allowed to rank selections. Temporal differences in rotation location and type may be confounded by student rankings or their desired placement location.

Using the EPA framework with ES scales was novel for preceptors in the UIC experiential program. While EPAs and ES scales are meant to use common language and focus on dichotomous decisions, a novel assessment may lead to inconsistencies and lower inter-rater reliability [8,9]. However, the large sample size of students across the multiple graduating classes will have reduced the noise within the analysis.

The translation of EPAs to a traditional grading system has yet to be defined, and most schools continue to use traditional assessment methods in combination with an ES rating [3,8]. However, at the UIC, these assessments are used to determine a student’s letter grade. Preceptors may have elevated the ES rating in order for a student to receive the letter grade they believed the student deserved.

### 4.3. Future Directions

Further evaluation of the ES scale and EPA-based assessment mechanisms will be needed to support their implementation. Additional research questions include how to incorporate multiple raters of EPA performance across different settings, ideal grading mechanisms to align with EPA use, how institutions can build quality assurances for this type of assessment, and what types of remediation may be needed in this model to support learners who are struggling. Technological advances in the documentation of multiple assessors for a learner taking EPAs with an ES scale show promise [28].

## 5. Conclusions

EPAs are an initial step towards functionalizing competency-based assessment in the workplace. Paired with ES scales, EPAs can be used as an assessment framework to determine the level of supervision a learner needs to perform a professional task. This study sought to highlight the longitudinal integration of EPAs into a pharmacy experiential curriculum and how it demonstrates student growth over time. The results documented consistent growth and suggest that this assessment framework can be feasibly implemented.

## Figures and Tables

**Table 1:** Jarrett Entrustment–Supervision Scale.

Level	Entrustment–Supervision Statement
1a	I would not trust the learner to perform or even observe this task. The learner lacks the professional behavior, knowledge, and related skill to perform or even observe this task.
1b	I would trust the learner to thoughtfully observe (but not perform) this task. The learner has foundational knowledge and skill about the task.
2a	I would trust the learner to perform this task WITH the preceptor and under full supervision. The learner will require direction, guidance and help during their performance of the task.
2b	I would trust the learner to perform this task under full supervision and the preceptor ready to step in, as needed. The learner is new in performing the task alone and guidance should be immediately available during the task.
3a	I would trust the learner to perform this task with on-demand, nearby preceptor supervision and ALL findings and work are checked immediately afterward.
3b	I would trust the learner to perform this task with on-demand, nearby preceptor supervision and KEY findings and work are checked immediately afterward.
3c	I would trust the learner to perform this task with on-demand, remote preceptor supervision and findings and work is audited soon afterward.
4	I would trust the learner to perform this task independently and unsupervised.
5	I would trust the learner to perform this task independently as well as to supervise and teach other learners.
Not Observed	Not applicable to this practice setting.

**Table 2:** Student demographics.

Characteristic		Class of 2022 (n = 196)	Class of 2023 (n = 176)	Class of 2024 (n = 186)
Gender, n (%)	Male	67 (34%)	69 (37%)	79 (45%)
	Female	129 (66%)	117 (63%)	97 (55%)
Age, Mean (Range)	Years	23 (19–42)	24 (19–43)	24 (19–49)
Residence, n (%)	Illinois	144 (73%)	143 (77%)	135 (77%)
	Other US State	47 (24%)	35 (19%)	41 (23%)
	International	5 (3%)	8 (4%)	0 (0%)
Highest Degree on Acceptance, n (%)	Associate	13 (7%)	26 (14%)	20 (11%)
	Bachelor	159 (81%)	116 (62%)	130 (74%)
	Master	4 (2%)	8 (4%)	8 (5%)

## Data Availability

The data presented in this study are available on request from the corresponding author due to privacy concerns.

## Abbreviations

The following abbreviations are used in this manuscript:

AMC	Academic Medical Center
APPE	Advanced Pharmacy Practice Experience
ES	Entrustment–supervision
EPA	Entrustable professional activity
IPPE	Introductory Pharmacy Practice Experience

## Appendix A. Entrustable Professional Activity Integration at the University of Illinois Chicago Retzky College of Pharmacy

**Table A1:** University of Illinois Chicago Retzky College of Pharmacy experiential course curriculum and descriptions.

Experiential Course	Course Description	EPA Utilization
PHAR 411: Introduction to Pharmacy Practice (Skills lab-based course)	Experiential core course offered in the fall semester to first professional year PharmD students. Introduces students to the practice of pharmacy through a combination of lectures, simulations, and a one-week off-site IPPE in an assigned pharmacy environment. Lectures and simulated activities cover communication skills with various audiences, interprofessional interactions, the tenets of professionalism, and medication dispensing, administration, and counseling. Students are expected to learn and recall basic drug information details from a provided list of prescription and non-prescription medications.	Pharmacy Practice Week reflection on EPA utilization at their practice site
PHAR 412: Community IPPE	Required experiential course where students are provided with an overview of contemporary pharmacy practice in a community setting. Develops student knowledge of the role of the Community Pharmacist through various lectures, workshops, and activities. Students also engage in a 6-week IPPE experience at a community pharmacy site where they gain valuable working knowledge of the community pharmacy environment and the roles and responsibilities of the pharmacy personnel. The goal of this experience is to engage students in an environment that will enable them to observe and develop the skills necessary to practice in a community setting. At the conclusion of this experience students should be comfortable with interpreting a prescription, filling a prescription, and providing patient counseling at a beginner’s level under pharmacist supervision.	Mid-point and final assessments by preceptor Mid-point and final self-assessment by student
PHAR 413: Hospital IPPE	Required experiential course where students are provided an overview of contemporary pharmacy practice in a hospital setting. Students spend most of their time engaged in actual (off-site at a hospital pharmacy) or simulated (on-site) hospital pharmacy practice activities. The goal of this course is to expand student understanding of the medication use process in a hospital setting, roles and responsibilities of pharmacists and technicians, as well as an introduction to interprofessional team dynamics. At the conclusion of this course, students should be comfortable with interpreting a medication order, correctly compounding an intravenous medication, verifying, and checking the accuracy of a filled medication order prior to dispensing, and communicating with members of the hospital team. Goals are expected to be completed under pharmacist supervision.	Mid-point and final assessments by preceptor Mid-point and final self-assessment by student
PHAR 414: Introduction to Patient Care (Skills lab-based course)	Fourth of six experiential courses required to complete the PharmD program. Students are introduced to the skills necessary to provide direct patient care. The goal of this course is to develop the skills necessary for communication of a pharmacotherapeutic recommendation both verbally and in writing. Upon course completion, students should be comfortable with conducting a medication history, performing medication reconciliation, optimizing pharmacotherapeutic regimens, presenting patient cases, and documenting in the Subjective/Objective/Assessment/Plan (SOAP) note format.	EPA surveys are utilized for student self-reflection after each simulation lab
PHAR 515: Institutional/Hospital IPPE	Designed to aid third year pharmacy students with the transition to APPEs. The primary setting for this course is in a direct patient care setting in a hospital where the student applies the didactic and early experiential coursework to the patient care setting.	Mid-point and final assessments by preceptor Mid-point and final self-assessment by student
PHAR 516: Ambulatory Care/Community IPPE	PHAR 516 is the Patient Care in an Ambulatory Care/Community setting course in the IPPE) series designed to aid third year pharmacy students with the transition to APPEs. The primary setting for this course is in a direct patient care setting in a hospital where the student will apply the didactic and early experiential coursework to the patient care setting.	Mid-point and final assessments by preceptor Mid-point and final self-assessment by student
Advanced Pharmacy Practice Experiences (APPE)	Students are required to complete seven APPEs consisting of four required core areas (Hospital (PHAR 603), Community (PHAR 602), Inpatient General Medicine (PHAR 604), and Ambulatory Care (PHAR 601)) and three elective areas. Each rotation is six weeks long and across different health systems for a broad range of direct and indirect patient care experiences.	Mid-point and final assessments by preceptor Mid-point and final self-assessment by student

**Table A2:** Core EPAs for pharmacy graduates mapped to UIC IPPE and APPE rotations for assessment.

	IPPE				APPE			
	Community (PHAR 412)	Hospital (PHAR 413)	Ambulatory Care (PHAR 516)	Hospital Inpatient (PHAR 515)	Community (PHAR 602)	Hospital (PHAR 603)	Ambulatory Care (PHAR 601)	Inpatient Medicine (PHAR 604)
Patient Care Provider Domain								
Collect information to identify a patient’s medication-related problems and health-related needs	x	x	x	x			x	x
2. Analyze information to determine the effects of medication therapy, identify medication-related problems, and prioritize health-related needs			x	x			x	x
3.. Establish patient-centered goals and create a care plan for a patient in collaboration with the patient, caregiver(s), and other health professionals that is evidence-based and cost-effective			x	x			x	x
4. Implement a care plan in collaboration with the patient, caregiver(s), and health professionals			x	x			x	x
5. Follow-up and monitor a care plan			x	x			x	x
Interprofessional Team Member Domain								
6. Collaborate as a member of an interprofessional team	x	x	x	x	x	x	x	x
Population Health Promoter Domain								
7. Identify patients at risk for prevalent diseases in a population					x		x	
8. Minimize adverse drug events and medication errors	x	x			x	x		
9. Maximize the appropriate use of medication in a population	x	x			x	x		
10. Ensure that patients have been immunized against vaccine-preventable diseases					x		x	
Information Master Domain								
11. Educate patients and professional colleagues regarding the appropriate use of medications	x	x			x	x	x	x
12. Use evidence-based information to advance patient care					x	x	x	x
Practice Manager Domain								
13. Oversee the pharmacy operations for an assigned work shift	x	x			x	x		
14. Fulfill a medication order	x	x			x	x		
Self-developer Domain								
15. Create a written plan for continuous professional development	x							

## Appendix B. Modeled Response Level Mapped to Entrustment–Supervision Level

**Table A3:** Modeled response level mapped to entrustment–supervision level.

	Mapped Response Level	
Level	Linear Model	Ordinal Model
Not Observed	0	1
1a	1	2
1b	1.3	3
2a	2	4
2b	2.3	5
3a	3	6
3b	3.3	7
3c	3.6	8
4	4	9
5	5	10

## Appendix C. Preceptor Assessment of Student Performance in Entrustable Professional Activities

**Table A4:** Number of assessments by graduating class and by preceptors for each included EPA.

Number of Assessments by Graduation Year		
Graduation Year	Students (n)	Assessments per Student (n)
2022	190	8748
2023	163	1887
2024	156	1791
**Number of Assessments per EPA by Preceptors**		
**EPA**	**Assessments by Preceptors (n)**	
1	2110	
2	2134	
3	2030	
4	2013	
5	1981	
6	2158	

**Table A5:** Differences in ES level by type of experience and training environment as assessed by preceptors.

Comparator	EPA	Difference in ES Level	*p*-Value
Based on setting for any point in time			
Inpatient to Outpatient	1	−0.05	0.02
Inpatient to Outpatient	2	−0.09	<0.05
Inpatient to Outpatient	3	−0.11	<0.05
Inpatient to Outpatient	4	−0.12	<0.05
Inpatient to Outpatient	5	−0.10	<0.05
Inpatient to Outpatient	6	−0.07	<0.05
AMC to non-AMC	1	−0.07	0.01
AMC to non-AMC	2	−0.12	<0.05
AMC to non-AMC	3	−0.10	<0.05
AMC to non-AMC	4	−0.11	<0.05
AMC to non-AMC	5	−0.12	<0.05
AMC to non-AMC	6	−0.09	<0.05

AMC = academic medical center.
